# Characterization of Fat Quality in Cow Milk from Alpine Farms as Influenced by Seasonal Variations of Diets

**DOI:** 10.3390/ani12040515

**Published:** 2022-02-19

**Authors:** Annalaura Lopez, Federica Bellagamba, Giovanni Savoini, Vittorio Maria Moretti, Donata Cattaneo

**Affiliations:** Department of Veterinary Medicine and Animal Science, University of Milan, Via dell’Università, 6, 26900 Lodi, Italy; federica.bellagamba@unimi.it (F.B.); giovanni.savoini@unimi.it (G.S.); vittorio.moretti@unimi.it (V.M.M.); donata.cattaneo@unimi.it (D.C.)

**Keywords:** alpine mountain milk, dairy cattle, fresh grass feeding, pasture grazing, fatty acids

## Abstract

**Simple Summary:**

Milk produced in Alpine farms under pasture- and-grass-based feeding systems is characterized by beneficial nutritional traits, which are linked to its high fat quality. In this study, milk samples collected in two alpine farms set in the Italian Piedmont region were analyzed together with samples of feedstuffs (pasture, fresh grass, concentrates and total mixed ration) furnished to cows during summer and winter, when the feeding strategy was modified. The results suggested a favorable fat composition of all samples analyzed, with some differences detected between the seasons and the farms. The milk obtained following a pasture- or fresh-grass-based feeding strategy (during summer) showed a higher fat quality, which was characterized by higher amounts of beneficial fatty acids. Particularly, a distinctive fat composition of milk samples coming from cows exclusively fed on pasture during the summer season was evidenced. The outcomes obtained in this study contributed toward evaluating and promoting alpine dairy products from the Alpine region as products associated with an added value, with beneficial effects for both producers and consumers.

**Abstract:**

The production systems linked to mountain animal husbandry have had an environmental, social and cultural role in recent years. Zootechnical systems based on feeding strategies, such as pasture grazing and grass-fed strategies, contribute to a significant increase in the relative amounts of favorable fatty acids (FAs) in animal products, indicating their ability to improve the long-term health of consumers. In this study, we compared different feeding strategies in two small mountain farms in the Piedmont Alpine region, Italy. Particularly, during the summer season, the two farms were distinguished by the exclusive employment of Alpine pasture (farm A), assumed as the best way to improve the quality of the FA profile in milk vs. the supply of daily fresh cut mountain grass plus a reduced implementation with hay and concentrates directly in the barn (farm B). The milk fatty acid profile was analyzed using gas chromatography. The results showed the high quality of alpine milk collected in the two farms. Even with some differences, particularly evidenced when comparing the summer diets, the milk FA profiles in farm A and farm B were favorable from a nutritional point of view in both seasons. Milk samples obtained using the exclusive employment of alpine grazing during summer were represented by an FA profile of higher quality (lower saturated FAs, higher branched FAs and monounsaturated FA, favorable n6/n3 ratio). However, milk obtained using the integrated strategy (fresh grass plus concentrates in the barn farm B) resulted in a more homogenous composition during the summer season, with a higher concentration of polyunsaturated FAs. These outcomes suggested that the integrated strategy, even if related to a lower ability in improving milk FA profile, could represent a valid and cost-effective alternative for mountain farmers to obtain an overall superior quality of milk, which was not strictly linked to the grazing practice. The multivariate analysis showed that information contained in the milk FA profile may provide a valuable tool that can distinguish mountain-grass-based diet.

## 1. Introduction

The mountain agricultural crisis in the Alpine region led to the loss of fundamental ecosystem services, including the loss of typical dairy products based on a pasture livestock system [1,2,3]. However, in recent years, the production systems linked to mountain animal husbandry have been acquiring an environmental, social and cultural role, leading to policies aimed at supporting them and to the promotion of a generational change in rural areas activities that were abandoned. The economic sustainability of mountain animal husbandry, which is mainly family-centered, is most related to touristic multiservice approaches and its generating interest in consumers for mountain dairy products considered as top-quality products, both for specific organoleptic and superior nutritional properties [4,5].

Recently, the European Union has granted the “mountain product” label [6] as a strategy to sustain local products from mountain farming systems and local development politics of alpine areas, giving an obvious geographical connotation that can generate added value for mountain products [7].

In dairy farming, low-input management systems, including feeding strategies based on pasture grazing, grass-fed and organic farming, are perceived by consumers as more sustainable, natural, healthy and respectful of animal welfare and biodiversity. Furthermore, dairy products from these rearing systems are known to possess an added value because of their favorable nutritional characterization and their high environmental sustainability, and are associated with authenticity features linked to their origin and traditional production processes [8].

The milk fatty acid (FA) profile changes very quickly following changes in the feeding regime, affecting rumen microbial biohydrogenation (RBH) and mammary gland activity in cows. Modifications of the housing system (outdoor and indoor) and the feeding strategy (grazing and integration) in mountain and alpine farms are strictly linked to the seasonal changes in climatic conditions and feed availability, which are considered critical factors that affect the chemical composition of milk in ruminants [9]. Farming systems characterized by grazing and/or the supply of a reduced amount of conserved forage contributes to a significant increase in the relative amount of n3 FA in milk, mainly represented by α-linolenic acid (18:3n3), given that fresh grass contains about 1–3% FA/DM and 50–75% of these FAs are represented by α-linolenic acid [10]. Moreover, together with the n3 FA series, fresh grass feeding and pasture grazing contribute to conveying a beneficial fatty acid composition to milk that involves a favorable composition in saturated/unsaturated FAs, *cis* and *trans* monounsaturated FAs (MUFAs), odd- and branched-chain FAs (OBCFAs) and polyunsaturated FAs (PUFAs), including conjugated linoleic acid (CLA) [11,12,13,14]. Particularly, the botanical composition of alpine pasture, which is mainly related to altitude, is known to induce significant modification in the milk FA profile, increasing favorable FAs and diminishing the unfavorable FA as a function of the elevation of the pasture [10,15,16].

Branched-chain fatty acids (BCFAs) in their *iso* and *anteiso* forms represent a lesser component of milk (about 2–3% of total fatty acids), but they are recognized as important bioactive components since their positive role in gastrointestinal microbial ecology and their cytotoxicity might be compared with CLA. Increasing the forage:concentrate (F:C) ratio resulted in a higher proportion of *iso*14 and *iso*15 BCFAs in cow milk, which was related to the effect of diet on the *iso* form content in rumen bacteria [17,18]. In addition, the odd-chain FAs (15:0, 17:0, 17:1) are generally higher in pasture cow milk and cheese and their concentration is influenced by different pasture vegetation types [15,16]. Some of the abovementioned FAs could be interesting reliable chemical markers in chemometric approaches provided for the discrimination of the origin of milk fat, contributing to the characterization and protection of typical dairy products [19,20].

The aim of the current study was to evaluate the variation in milk fat quality as affected by seasonal variation of the housing and feeding system in two Alpine farms in Piedmont (Italy), during summer and winter. The study was conducted under the usual conditions of farm management of herds in the Alpine region rather than under controlled experimental conditions.

## 2. Materials and Methods

### 2.1. Farming Conditions and Diets

The trial was carried out in the Northern Alpine area of Piedmont, a northwestern region of Italy, during two subsequent seasons, summer (August–September 2018, 51 days) and winter (January–March 2019, 77 days). Two alpine farms were involved in the trial, selected as representatives of the farming systems of interest that were typical of the alpine areas. Both farms processed raw milk in their dairy for cheese making; particularly, during summer, farm A transformed milk directly on mountain pasture.

The herd in farm A consisted of 40 multiparous dairy cows (Brown Swiss, Simmental and cross-bred cows). Farm A was set at 520 m above sea level and during winter (from October to late May), cows were housed indoors and fed a total mixed ration (TMR), consisting of alfalfa, mixed grass hays and concentrates. During summer, dairy cows were fed exclusively natural Alpine pasture, grazed at an altitude ranging from 1500 to 2000 m above sea level (100% of supplied DM).

Farm B was set in an alpine valley at 1300 m above sea level. The herd in farm B consisted of 43 multiparous Brown Swiss dairy cows housed indoors all year long. During winter (from October to late May) cows were fed a TMR consisting of meadow hay and concentrates. In summer, during the daytime, they were fed daily-cut fresh grass (31.7% of supplied DM), harvested in the grassland meadows of the surrounding Alpine valley (1250 to 1750 m above sea level), while in the evening, a TMR consisting of alfalfa hay and concentrates (58% of supplied DM) was furnished to the cows. Moreover, in this farm, cows were supplied with additional concentrates (10.2–10.3% of supplied DM) directly in the milking parlor in both summer and winter. The ingredient compositions of the summer and winter diets fed in the two dairy farms is reported in Table 1.

### 2.2. Feedstuffs and Milk Sampling

In both farms, dietary feedstuffs and bulk-tank milk samples were collected once every 7 days (8 sampling times for each season during the study). In farm A, summer pasture grass was harvested in six different randomly selected areas of the pastureland, cut to about 5 cm in height, in line with the feeding behavior of cows. In order to avoid the collection of grass discarded by animals, the sampling procedure was performed in pasture areas where cows had not grazed yet. In farm B, summer daily-cut fresh grass was sampled before being offered to cows. In both farms, the samples of tufts of pasture or daily-cut fresh grass were collected in triplicate and mixed in a pool in order to obtain a representative sample (500 g); they were then stored frozen under vacuum until analyses were performed. Bulk milk samples were collected directly from the farm in 50 mL plastic tubes after stirring the content of the tank. Hay and other feedstuffs and milk bulk samples were transported to university laboratories under refrigeration and stored at −20 °C until analysis.

**Table 1:** Compositions of the winter and summer diets in the two farms involved in the study. Each ingredient is expressed as kg/head per day on an as-fed basis. The components of the diets (fresh grass, concentrate, total mixed ration, TMR), in bold, are presented as a percentage of the total supplied dry matter (%SDM). AS—farm A summer, BS—farm B summer, AW—farm A winter, BW—farm B winter.

Summer			Winter			
AS	BS		AW		BW	
**Only pasture** **100% SDM**	**Fresh Forages (31.7% SDM)**					
	Daily-cut fresh grass	35 kg				
	**TMR (58% SDM)**		**TMR (100% SDM)**		**TMR (89.8% SDM)**	
	-Alfalfa hay	6 kg	-Alfalfa hay	10 kg	-Meadow hay	12.5 kg
	-Concentrate A *	5 kg	-Mixed grass hay	4.5 kg	-Concentrate A *	5.5 kg
	-Commercial concentrate **	3 kg	-Concentrate (CP 18%)	4.5 kg	-Commercial concentrate **	3.5 kg
	-Molasses	0.5 kg	-Flaked corn	2 kg	-Molasses	1 kg
	Total TMR	14.5 kg	-Beet pulps	1 kg	Total TMR	22.5 kg
			-Straw	1 kg		
			-Whey	12 kg		
			Total TMR	35 kg		
	**Concentrate at Milking** **(10.3% SDM)**				**Concentrate at Milking** **(10.2% SDM)**	
	Concentrate B ***	2.5 kg			Concentrate B ***	2.5 kg

* Concentrate A: corn, barley and sodium bicarbonate. Analytical composition (% as fed): crude protein 8%, crude fibre 2.47%, crude fat 3.50%, ashes 1.87% and Na 0.05%. ** Commercial concentrate: soybean meal (dehulled), sunflower meal (decorticated), soybean (toasted), corn gluten feed, wheat bran, sunflower meal, calcium carbonate, sodium bicarbonate, dicalcium phosphate and sodium chloride. Analytical composition (% as fed): crude protein 33%, crude fibre 10.5%, crude fat 3.70%, ashes 9.8%, Na 0.57% plus trace elements and vitamin integration. *** Concentrate B: corn, wheat flour middlings, barley, soybean meal (dehulled), soybean (toasted), sunflower meal (decorticated), wheat bran, soybean hulls, dicalcium phosphate, sodium chloride, calcium carbonate, sodium bicarbonate and magnesium oxide. Analytical composition (% as fed): crude protein 14.2%, crude fibre 4.9%, crude fat 4.50%, ashes 6.5%, Na 0.3% plus trace elements and vitamin integration.

### 2.3. Composition and Fatty Acid Content of Dietary Feedstuffs

AOAC official methods [21] were used to determine dry matter (DM), crude protein (CP), crude fat (CF) and ash according to the method of Van Soest et al. [22] to determine the neutral detergent fiber (NDF) and acid detergent fiber (ADF) of pasture, fresh daily-cut grass, concentrates and TMR samples. Lipids in feedstuff samples were extracted using ether extraction with petroleum ether. Fatty acid methyl esters (FAMEs) were prepared using base-catalyzed esterification with KOH/methanol and identified using gas chromatography and flame ionization detection using a TRACE™ 1300 chromatograph equipped with a TR-FAME column (Thermo Fisher Scientific Waltham, MA, USA). For the FA composition of fresh forage, we focused on the six main FAs that constituted the greatest majority of the total FA in grass: palmitic (16:0), palmitoleic (*cis9*-16:1), stearic (18:0), oleic (*cis9*-18:1), linoleic (*cis9cis12*-18:2) and α-linolenic (18:3n3) acids.

### 2.4. Fatty Acid Profile of Milk

Lipids of milk samples were extracted and quantified by means of liquid–liquid extraction with chloroform and methanol (2:1) according to the method of Folch et al. [23]. An aliquot of 40–50 mg of extracted lipids was employed for the fatty acid (FA) profile determination through the methyl-esterification in an alkalyne environment using sodium methoxide in methanol 1M, as described by Christie [24]. This procedure allowed for the methyl-esterification of FAs linked to glycerol molecules in triacylglycerides (TAGs) of milk fat globules. FAMEs prepared in this way were identified using gas chromatography and flame ionization detection using a TRACE™ 1300 chromatograph equipped with a TR-FAME column (120 m, 0.250 mm id, 0.25 µm film thickness) from Thermo Fisher Scientific (Waltham, MA, USA). The programmed oven temperature started from 45 °C, was held for 8 min, increased at a rate of 10 °C/min up to 173 °C, and this temperature was kept for 47 min. Finally, a temperature gradient of 4 °C/min was set until reaching the final oven temperature of 220 °C, which was held for 30 min. FAMEs were identified by comparing peak retention times with FA standard mixtures and pure standard purchased from Sigma-Aldrich (St. Louis, MO, USA) and Larodan (Solna, Sweden) and analyzed under the same analytical conditions. Correction factors for the GC-FID response for FAMEs from C4 to C12 were applied [25]; then, fatty acid amounts were expressed as g 100 g^−1^ of the total FA.

### 2.5. Data Analysis

Data analysis was performed using JMP Pro 15 from the SAS Institute (Cary, NC, USA). Milk FAs (dependent variables) were analyzed by means of a two-way ANOVA, considering the farm (F), the season (S) and their interaction (F × S) as factors influencing data variability. Tukey’s HSD was used for post hoc comparisons, considering the observed differences as significant when *p* < 0.05 (*), *p* < 0.01 (**) and *p* < 0.001 (***). In order to reduce the dimensionality of the original data matrix and to observe the distribution of the samples, a principal component analysis (PCA) was performed as an unsupervised multivariate test, which was directed to detect the presence of eventual clusters or relationships between samples. Before developing the PCA, a low-level data fusion [26] was performed between the data on feedstuff gross compositions and FA profile and the milk FA profile, leading to a new data matrix consisting of 32 samples × 69 variables. Data were auto-scaled [27] before performing the multivariate analysis in order to reduce the bias due to the influence of the parameters related to higher measure units. The score plot and the loading plot obtained by plotting PC1 vs. PC2 were used for data interpretation.

## 3. Results and Discussion

### 3.1. Diets Composition

The chemical composition and the FA profile of summer and winter diets supplied in the two Alpine mountain farms are reported in Table 2.

The outcomes obtained by the proximate analysis of alpine grass supplied to cows during the summer season (Table 2) were comparable to those previously obtained by other authors who studied the composition of meadows collected in different Italian alpine areas [15,28,29,30,31,32]. Particularly, the alpine grass analyzed in this trial showed an average crude protein (CP) content ranging from 12.53% DM (farm A) to 16.12% DM (farm B) and an NDF content ranging from 60.21% DM (farm B) to 67.26% DM (farm A). Since the CP and NDF proportions are strongly related to the quality of forages [29], the high values reported in this trial for these two parameters suggested an optimal quality of the meadows used as a forage source for dairy cows in the farms studied during the considered production seasons. Gorlier et al. [28] detected negative and positive correlation coefficients between the phenological phases of the plants and the CP and NDF contents of the forages, respectively. The trend detected in this study for the grass collected in farm B (lower NDF and higher CP than in farm A) could suggest an average earlier phenological stage of the herbal essences present in the grassland meadows used in farm B.

Considering the total daily rations employed in summer, diets greatly differed between the two farms (Table 1 and Table 2). While in farm A, cows were fed grass only on pasture with no integration, in farm B, the diet was based on daily cut fresh grass integrated with a TMR (alfalfa hay and concentrates) plus further concentrates furnished in the milking parlor. Consequently, as expected, the values of NDF and ADF were higher in the whole diet of farm A (respectively, 67.26 and 36.63% DM in farm A vs. 42.03 and 27.55% DM in farm B) due to the higher content of forages included in the total daily ration (100% DM in farm A vs. 56% DM in farm B).

The fatty acids (FAs) composition showed interesting differences between the pasture grass supplied in the two farms during summer. In particular, pasture grazed in farm A showed higher values of oleic (18:1n9) and linoleic (18:2n6) acid, while fresh grass collected in farm B showed higher values of α-linolenic acid (18:3n3). The differences observed in the FA compositions of the two summer grasslands could probably be related to several natural factors, such as the presence of different botanical families, different altitudes of pastures and distinct growth phases of the plants [29,33]. However, the amounts detected for oleic (4.43–12.91%), linoleic (16.28–21.46%) and α-linolenic (27.80–38.27%) acids in pasture grass from both farms were comparable to those detected by [34] in alpine pastures collected in the Piedmont region during summer, ranging from 6.01 to 13.5% for oleic acid, 18.1 to 23.9% for linoleic acid and 29.7 to 46.6% for α-linolenic acid.

Since fresh grass only represented one-third (30%) of the diet supplied to dairy cows in farm B during summer, the FA composition of the total daily ration furnished in this farm was found to be qualitatively different compared with farm A. Higher levels of saturated (palmitic acid 16:0 and stearic acid 18:0), monounsaturated (oleic acid) and n6 PUFA (linoleic acid) were observed for the total daily ration of farm B, where the FAs were highly representative of the concentrates furnished with the diet.

Winter diets adopted in the two mountain farms were comparable, with both consisting of TMR with hay and different concentrates, which were substantially different from the summer diets based on fresh grass (Table 1). Both the gross composition and the FA profile of the total daily rations furnished in farm A and farm B during winter showed smaller differences between them than the summer counterparts did (Table 2). When compared with the summer diets, the greatest differences were observed for the FA profile, with lower levels of α-linolenic acid (5.68 and 6.82% in farm A and farm B, respectively) and higher levels of oleic (25.1 and 26.90% in farm A and farm B, respectively) and linoleic acid (47.75 and 42.27% in farm A and farm B, respectively) detected in winter diets. The differences observed were due to the supply of concentrates at higher proportions during the winter season (reaching an F:C ratio of 65:35 in farm A and 50:50 in farm B). The greatest difference was observable between the summer and winter diets employed in farm A, where no integration with concentrates was performed during the summer season.

### 3.2. Milk Composition

Milk samples collected from the two farms showed average milk fat concentrations ranging from 3.7 ± 0.6% (AS) to 3.2 ± 0.2% (BS) during summer and from 3.0 ± 0.8% (AW) to 3.1 ± 0.8% (BW) during winter. Results obtained for the FA profile of milk fat are reported in Table 3.

The correlation coefficients between the fiber-related parameters of the diets (NDF, ADF content and F:C ratio) and the concentrations of FAs detected in the milk are presented in Table 4.

#### 3.2.1. Saturated Fatty Acids (SFAs)

Overall, no statistical differences were detected for total SFAs between the two seasons for each farm. However, focusing on the summer samples, we detected significantly lower amounts of SFAs in farm A, where cows were fed just by grazing (62.21% SFAs in AS samples vs. 67.43% in BS samples). The differences were mainly related to higher amounts of short- and medium-chain FAs from 6:0 to 14:0 in farm B. In contrast, long-chain FAs from 18:0 to 24:0 were found higher in AS than in BS milk. According to this, we observed negative correlation coefficients (Table 4) between the F:C ratio, the NDF and the ADF amount of the diet and the amount of milk 6:0–14:0 FAs, with correlation coefficients reaching the highest values (near to −0.80) for 10:0, 12:0 and 14:0. In contrast, the correlation coefficients between the fiber-related parameters of the diet and the amount of long-chain SFAs (from 18:0 to 24:0) in milk were positive, even if represented by lower values (<0.80).

These results are in agreement with the scientific literature asserting that pasture practice and diets represented by high F:C values can increase the concentration of 18:0 and decrease the concentration of hypercholesteroleamic FAs (12:0, 14:0 and 16:0) in milk [12,36]. Revello Chion et al. [34] observed that cows fed with fresh grass produced milk characterized by a favorable FA composition compared with animals fed with dry diets, especially in the summer season, with reduced levels of saturated FAs. This outcome is very interesting for consumers since the medium-chain SFAs 12:0, 14:0 and 16:0 were shown to be associated with adverse effects on indicators of cardiovascular disease and their excessive consumption is related to an increased risk of atherosclerosis, hyperlipidemia, obesity and coronary heart diseases in humans [36].

#### 3.2.2. Odd- and Branched-Chain Fatty Acids (OBCFAs)

Increasing the F:C ratio in the diet of dairy cows generally produces an increase of milk odd- and branched-chain fatty acids (OBCFAs), particularly the branched form [17,37]. This modification is related to a shift in the proportion of the rumen bacterial population toward the growth of cellulosolytic bacteria, which was enhanced by increasing the proportion of forages in the diet. Several authors reported a positive correlation between the NDF content of the diet and milk OBCFA content [17,37,38]. Accordingly, in this investigation, we observed positive correlations between the dietary fiber content and the amounts of 17:0 and several branched-chain fatty acids (BCFAs), with the highest correlation coefficients (>0.80) observed between the F:C ratio of the diet and the BCFA content in milk (Table 4).

We found total OBCFAs to be significantly higher in the AS samples (4.97%) than in all the other groups. Particularly, the BCFAs that reached the higher concentration in AS samples were 17:0, *iso*14, *iso*15, *anteiso*15, *iso*16 and *anteiso*17. These FAs are associated with a particular significance, especially since

- They have been recognized as biomarkers of the rumen functionality, with their proportions strictly related to the ruminal microflora system (mainly, differences between cellulosolytic and amylolitic bacteria);
- They are known as bioactive food components since they display healthy functional properties that are comparable with that of greater debated CLAs isomers [36,39].

In this work, we observed and confirmed that the employment of pasture represents an effective strategy to maximize the presence of beneficial functional FAs in milk products. The highest values for the BCFAs (3.24%) were found in farm A during summer (AS) when cows were fed exclusively by pasture; this result was significantly different from all other milk samples. In contrast, no differences were detected for BCFAs between summer (BS) and winter (BW) samples in farm B; this can be noticeably related to the highest similarity between the feeding regime chosen in this farm, where the integration strategy with concentrates was practiced in both seasons (summer and winter). Similarly, the amount of total OBCFAs was found to be higher in farm A than in farm B in both seasons, recording the highest amount in AS samples.

#### 3.2.3. Unsaturated Fatty Acids (UFAs)

Total monounsaturated fatty acids (MUFAs) content was higher in milk from farm A than in farm B in both seasons. The main MUFA accounting for this difference was oleic acid—*cis*9-18:1—which reached the highest amount (24.64%) in AS samples. We observed a positive correlation index between the F:C ratio, the NDF and the ADF amount of the diet and the amount of oleic acid in milk (correlation coefficient around 0.80, Table 4). Chilliard et al. [12] showed that oleic acid can be higher in pasture milk despite the low amount of this fatty acid in the pasture grass. Leiber et al. [32] suggested that this phenomenon could be related to the fact that pastures are usually enriched in α-linoleic acid—18:3 n3—which, after the rumen biohydrogenation processes, is converted to stearic acid and successively to its unsaturated counterpart oleic acid via mammary Δ9-desaturation. Moreover, it was suggested that the alpine pasture conditions could lead cow metabolism to a higher depletion of the adipose tissue (enriched in oleic acid) for milk FA synthesis, resulting in a higher amount of oleic acid in pasture milk [12].

Palmitoleic acid—16:1—was found in significantly lower amounts in AS milk (0.64% vs. 1.24–1.55% in other samples). We hypothesize that the lower amounts of palmitic acid supplied with the diet in farm A during summer (11.75%, Table 2) led to the lower availability of this FA in the mammary gland for the desaturation operated by the Δ-9 desaturase, which adds a *cis*9-double bond in the carbon chain of saturated FA from C10 to C19, leading to the formation of palmitoleic from palmitic acid [12]. However, the range of amounts (0.64–1.55%) detected for this FA was comparable to that found in the literature for cows fed freshly cut grass indoors or at pasture [19,40].

MUFAs with the double bond set in the *trans* configuration did not show statistically significant differences between the groups. It has been most often reported that pasture- and forage-based feeds increase the levels of *trans* FAs in milk, mainly *trans*18:1, simultaneously with increased levels of conjugated isomers of linoleic acid (CLAs) [12,41,42]. Indeed, vaccenic acid—*trans*11, 18:1—is a common intermediate in the rumen biohydrogenation (RBH) of linoleic and α-linolenic acid in the rumen. The RBH averages 80% for linoleic acid and 92% for linolenic acid [43]. In the RBH pathway, vaccenic acid is reduced to stearic acid but, due to the low rate of this reaction, it accumulates in the rumen, becoming available for absorption and transport to the mammary gland, where it is transformed to rumenic acid—*c9t11*, 18:2 CLA—by the Δ9-desaturase. Since the hydrogenation of *trans*-18:1 constitutes the limiting step for the full hydrogenation of unsaturated C18 FA, vaccenic acid frequently accumulates in the rumen. The comparable amount of vaccenic acid recorded in milk from the two farms and the two seasons in this study supported the consideration of this FA as a marker of high-forage diets (associated with a high F:C ratio), and not just linked to pasture practice. It can be observed that vaccenic acid amount showed a huge intra-group variability in AS samples, influencing the lack of significance when performing the statistical comparisons. Dewhurst et al. [42] had previously suggested that a higher variation between individual animals can be observed under grazing conditions than with a total mixed ration (TMR), a phenomenon that could potentially mask differences in experiments carried out on grazing systems in which we expected to find the highest level of vaccenic acid.

Similarly, no statistical difference between the amount of α-linolenic acid in milk delivered by the two farms in the two seasons was observed. The content of this fatty acid is known to vary according to botanical composition and maturation stage of the grassland, which would explain the higher proportion variability of α-linolenic acid, especially during the summer season, when cows were fed using pasture or freshly cut grass indoor [19].

One of the main intermediates of the rumen biohydrogenation (RBH) toward α-linolenic is rumenic acid (*c9t11*-CLA). During the RBH, a small proportion of the CLA is nonetheless absorbed in the intestine and secreted into milk through the mammary gland. The rumenic acid synthesis mainly occurs (probably more than 75%) in the udder, in proportion to the amount of vaccenic acid formed in the rumen [12]. We found *c9t11*-CLA in higher amounts in summer samples in both the farms (1.12–1.31%) than in the winter samples (0.73–0.78%). These outcomes agree with the knowledge that the consumption of fresh grass (both with grazing and furnishing it in the barn) is related to higher PUFA intake, higher RBH processes and a higher amount of the intermediates migrating to the mammary gland for the synthesis of milk FA [12]. However, the highest amount was detected in BS samples, even if the diet was associated with a higher proportion of concentrates compared with farm A. It is known that milk CLA composition in cows led to pasture are very variable due to the pasture composition (young grass contains higher 18:3n3) and the botanical origin of meadows. In this study, the α-linolenic acid content was higher in fresh cut grass from alpine meadows (35.74%, farm B) than in pasture grass (27.41 g/100 g FA, farm A), showing a trend with highest values in July (45.8%) and the lowest values when season advanced (late September, 22.97%) (seasonal data not reported). Furthermore, the dietary factors influencing the CLA concentrations in milk (18:2n6 and 18:3n3 precursors, forages and starchy sources) are interrelated and interactions between them can lead to wide variations (up to 4%) in CLA in pasture milk [44].

Linoleic acid was found in higher amounts in BS (2.73%) than in AS (1.38%) milk. Negative correlation indexes were observed between the F:C ratio, NDF and ADF amount of the diet and the total n6 FA content, particularly linoleic acid, showing correlation coefficients ranging from −0.75 to −0.86 (Table 4). The difference observed in the linoleic acid amount was directly linked to the higher n6/n3 ratio found in BS (3.17) than in AS (1.67) samples. The n6/n3 ratio in milk essentially depends on concentrations of linoleic (n6) and α-linolenic (n3) acids [45]. The n6/n3 ratio, well known as a healthy index to evaluate the quality of food fat, in dairy products can be improved (i.e., decreased) by shifts in cows diet, particularly enhancing the consumption of fresh grass, such as it happens in the production of alpine milk [32,45]. Accordingly, the n6/n3 ratio was already shown to reach values more than two times higher in conventional milk compared with extensive or pasture-based milk [46,47]. All the values recorded for n6/n3 in this study can be counted as favorable. They ranged from 1.67 to 3.33, falling in the suggested range of 1–4, which is considered optimal from a functional perspective in order to prevent many cardiovascular diseases in consumers [48]. Nonetheless, it is notable that milk produced by feeding cows just through the grazing practice was represented by the best n6/n3 balance.

### 3.3. Multivariate Analysis

**Figure 1:**
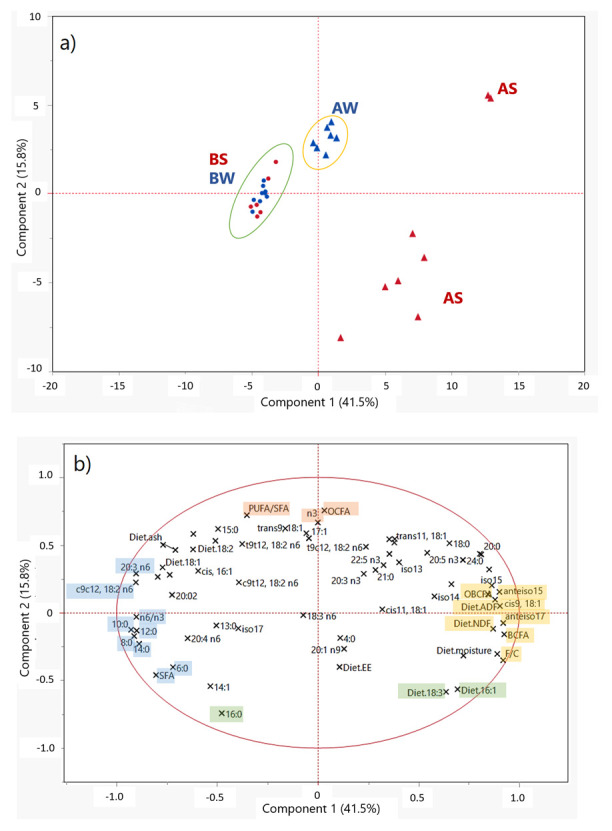
(**a**) PCA score plot. Legend: triangles—milk collected from farm A; points—milk collected from farm B; red—summer samples; blue—winter samples. (**b**) PCA loading plot.

In Figure 1, the scores plot (A) and the loading plot (B) of a PCA that obtained the first (PC-1) and the second (PC-2) principal components (PCs) are reported. The two PCs accounted for 57.3% of the total variability in the 32 (samples) × 69 (analytical parameters) data matrix.

When observing the set of samples in the multivariate system (Figure 1a), we noticed that within the mountain dairy production systems evaluated, many qualitative differences were delivered to milk employing different livestock managing and feeding strategies. Both farms involved in this study can be considered as “low input farms”, carrying a low farming pressure toward animals and the environment. However, in the PCA scores plot, samples were separated into three main groups, with BS and BW samples overlapping and clustering; these were separated from milk samples from farm A. AS milk was clearly separated from all the other samples; the main reason for this discrimination was due to by the total absence of any integration or concentrates included during the summer season in farm A. The PCA of average milk FA contents supported results previously obtained, which showed a clear separation between TMR-fed and pasture-based milk [19,49].

The factors with the highest negative loading scores for the PC-1 (in light blue in Figure 1b) were some SFAs (10:0, 14:0, 12:0, 8:0, 20:0 and 6:0) and n6 FAs (namely, c9c12-18:2n6, 20:3n6 and 20:4n6). Milk samples characterized by negative scores for PC-1 were the ones collected in farm B (Figure 1a), which were associated with the highest proportion of concentrates, and thus of linoleic acid in the dietary ration (Diet.18:2 in Figure 1b). This outcome suggested that, even if most of the linoleic acid introduced with the diet was transformed by the biohydrogenation processes occurring in the rumen, a certain amount of this FA escaped this pathway and was delivered to the mammary gland during milk fat synthesis, leading to a higher concentration in milk fat and, consequently, to a higher n6/n3 ratio in the milk. On the other hand, samples associated with the highest positive scores for PC-1 were AS samples (Figure 1a). The variables with the higher weight in the positive direction of PC-1 (in yellow in Figure 1b) were the fiber-related parameters of the diet (F:C, Diet.NDF and Diet.ADF) plus the milk content of many OBCFAs (namely, *anteiso*17, *anteiso*15 and 17:0) and oleic acid (*cis*9-18:1). BCFAs are known to be strictly related to a ruminal environment enriched in cellulosolytic bacteria, whose presence increase in the rumen when the diet is represented by a high amount of fiber, thus by a high F:C ratio. Hence, we suggest that the feeding strategy chosen during summer in farm A, represented by the exclusive employment of alpine grazing, involved a rumen functionality leading to a higher representation of beneficial functional FAs in milk.

AW samples represented a third separated cluster in the PC space, which was characterized by an intermediate position over PC-1 but clearly separated from AS samples over the direction of PC-2. Particularly, AS samples were characterized by negative PC-2 scores, while AW samples were characterized by positive PC-2 scores. The variables associated with positive loadings for PC-2 (orange in Figure 1b) were the OCFA, n3 and PUFA contents of milk; in contrast, the variables related to negative loadings for PC-2 (green in Figure 1b) were the milk content of palmitic acid (16:0) and the amount of α-linolenic acid and palmitoleic acid of feedstuffs (Diet.18:3 and Diet.16:1, respectively). It was interesting to observe that the content of α-linolenic acid of the diet (Diet.18:3) and the proportion of this FA in milk (18:3n3 in Figure 1b) were negatively correlated following the direction of PC-2. This confirmed the knowledge that the rumen biohydrogenation processes affect most of the content of α-linolenic acid supplied to cows with the diet.

Nevertheless, the distribution of the scores in the PCA plot (Figure 1a) suggested that the exclusive use of grazing pasture in farm A during summer led to a lower homogeneity of milk quality during the sampling time in summer. The feeding strategy based only on pasture, even if related to an overall improvement of milk fat quality, was also associated with higher variability in the milk FA profile due to the less controlled conditions (higher seasonal variability of forages, possibility for cows to choose what to eat, etc.) that characterized the grazing practice.

## 4. Conclusions

Milk fat composition significantly contributes to the promotion of dairy products associated with an added value, especially when characterized by a favorable FA profile. This issue could be particularly important for mountain and alpine products since their high value, as generally perceived by consumers, can be supported by analytically assessed nutritional quality. The results obtained by this study supported the evaluation of alpine milk collected in the farms involved in the investigation as high-quality milk based on its fat composition. Even though there were some differences between the two farms, as particularly evidenced when comparing the summer diets, the milk FA profiles in farm A and farm B were favorable from a nutritional point of view (high amount of functional active FAs, such as BCFAs and CLAs, favorable n6/n3 ratio, etc.) in both the seasons. Particularly, milk samples obtained using the exclusive employment of alpine grazing (AS) were represented by an FA profile of greater quality, even if characterized by a higher variability due to the less controlled feeding conditions during grazing. On the other hand, milk obtained using the integrated strategy (fresh grass plus concentrates in the barn BS samples) resulted in a more homogenous composition during the summer season, with a higher concentration of PUFAs.

Reported results suggested how the supply of fresh grass directly in the barn might represent a valid alternative to alpine pasture, aiming to obtain increased quality of milk characterized by high homogeneity and being more feasible for farmers.

The limited number of farms involved did not allow for extending our conclusions to a broader context. However, our results supported the idea that information contained in the milk FA profile may be used to assess the fresh grass feeding. Further investigations extended to a higher number of farms with similar characteristics in the same geographical area could be taken into account in order to create a tool to support and enhance the consumers’ perceptions toward alpine milk from the Piedmont alpine region.

## Figures and Tables

**Table 2:** Chemical compositions and FA profiles of the summer and winter diets supplied in the two farms (mean values). The chemical composition (CP, EE, ash, NDF, ADF) is presented as the percentage of the dry matter (%DM), while the fatty acid composition is presented as a percentage of the total FA.

Summer Diets					
	AS	BS			
	Pasture (Mixed Pools)	Fresh Grass	TMR	Concentrate B	Total Daily Ration
%DM	38.13 ± 6.71	18.79 ± 2.39	63.55	88.7	51.95 ± 0.76
CP %DM	12.53 ± 0.75	16.12 ± 1.54	15.78	16.22	15.93 ± 0.49
EE %DM	2.67 ± 0.21	2.88 ± 0.25	2.26	2.97	2.53 ± 0.08
Ash% DM	5.11 ± 0.86	12.65 ± 3.60	7.01	7.02	8.80 ± 0.56
NDF %DM	67.26 ± 6.96	60.21 ± 2.67	36.04	19.84	42.03 ± 0.85
ADF %DM	36.63 ± 1.79	40.97 ± 2.50	23.93	6.6	27.55 ± 0.85
F:C Ratio	100	56:44			
C16:0	11.75 ± 0.69	12.07 ± 2.09	13.94	22.51	14.23 ± 0.66
C16:1	1.58 ± 0.16	2.11 ± 0.42	0.37	0.31	0.91 ± 0.13
C18:0	1.97 ± 0.35	1.75 ± 0.43	3.33	5.19	3.02 ± 0.13
C18:1	12.91 ± 2.80	4.43 ± 0.98	26.55	36.67	20.58 ± 0.31
C18:2	21.46 ± 1.19	16.28 ± 2.69	37.86	23.07	29.50 ± 0.85
C18:3	27.80 ± 6.51	38.27 ± 5.67	3.91	1.87	14.59 ± 1.80
**Winter Diets**					
	AW	BW			
	Total Daily Ration		TMR	Concentrate B	Total Daily Ration
%DM	61.67		71.53	88.7	73.28
CP %DM	14.69		13.74	15.25	13.89
EE %DM	1.59		2.34	4.45	2.56
Ash% DM	9.05		7.73	7.17	7.67
NDF %DM	58.48		46.55	20.93	43.94
ADF %DM	34.42		29.79	7.72	27.54
F:C Ratio	65:35		50:50		
C16:0	13.51		13.26	23.14	14.27
C16:1	0.55		0.44	0.19	0.41
C18:0	2.34		3.28	5.78	3.54
C18:1	25.19		25.41	39.98	26.90
C18:2	47.75		44.76	20.38	42.27
C18:3	5.68		7.49	0.97	6.82

AS—farm A summer, BS—farm B summer, AW—farm A winter, BW—farm B winter. CP—crude protein, EE—ether extract, NDF—neutral detergent fiber, ADF—acid detergent fiber.

**Table 3:** FA composition (g/100 g of FA) of milk samples collected from farms A and B during summer and winter. Data are presented as mean ± standard deviation. AS—farm A summer milk, BS—farm B summer milk, AW—farm A winter milk, BW—farm B winter milk.

FA	AS	BS	AW	BW		*sign*	
					F	S	S × F
Saturated Fatty Acids (SFAs)							
4:0	5.11 ± 0.67	4.91 ± 0.22	4.96 ± 0.17	4.89 ± 0.24	ns	ns	ns
6:0	2.46 ± 0.37 B	2.85 ± 0.36 A	2.57 ± 0.07 AB	2.70 ± 0.10 AB	ns	*	ns
8:0	1.35 ± 0.27 B	1.75 ± 0.19 A	1.53 ± 0.05 AB	1.68 ± 0.05 A	ns	***	*
10:0	2.44 ± 0.67 C	3.76 ± 0.30 A	3.03 ± 0.20 B	3.54 ± 0.12 AB	ns	***	**
12:0	2.70 ± 0.79 B	4.18 ± 0.23 A	3.35 ± 0.28 C	3.92 ± 0.14 AB	ns	***	**
14:0	9.16 ± 1.54 C	12.31 ± 0.15 A	10.65 ± 0.69 B	11.62 ± 0.30 AB	ns	***	**
16:0	27.42 ± 4.77	29.23 ± 0.91	26.40 ± 0.78	28.59 ± 0.41	ns	ns	ns
18:0	11.21 ± 2.63 A	8.20 ± 0.41 B	11.18 ± 0.77 A	10.31 ± 0.50 A	*	***	*
20:0	0.20 ± 0.05 A	0.16 ± 0.05 AB	0.19 ± 0.01 AB	0.15 ± 0.01 B	ns	**	ns
22:0	0.10 ± 0.04 A	0.06 ± 0.02 B	0.09 ± 0.01 AB	0.06 ± 0.00 B	ns	***	ns
24:0	0.07 ± 0.03 A	0.04 ± 0.02 B	0.06 ± 0.01 AB	0.04 ± 0.00 B	ns	**	ns
Total SFAs	62.21 ± 5.32 B	67.43 ± 0.85 A	64.01 ± 1.07 AB	67.51 ± 0.53 A	ns	***	ns
Odd- and Branched-Chain Fatty Acids (OBCFAs)							
13:0	0.08 ± 0.02 B	0.11 ± 0.02 A	0.08 ± 0.01 B	0.10 ± 0.01 AB	ns	**	ns
15:0	0.78 ± 0.21 B	1.10 ± 0.06 A	1.14 ± 0.07 A	1.03 ± 0.03 A	**	*	***
17:0	0.74 ± 0.09 A	0.52 ± 0.01 B	0.66 ± 0.06 A	0.51 ± 0.02 B	ns	***	ns
17:1	0.09 ± 0.04 C	0.05 ± 0.01 D	0.26 ± 0.04 A	0.17 ± 0.01 B	***	***	*
21:0	0.04 ± 0.01	0.04 ± 0.02	0.05 ± 0.06	0.03 ± 0.00	ns	ns	ns
Total OCFAs	1.73 ± 0.30 B	1.80 ± 0.06 B	2.19 ± 0.15 A	1.83 ± 0.04 B	***	*	**
*iso*13	0.04 ± 0.01	0.04 ± 0.00	0.04 ± 0.00	0.04 ± 0.00	ns	ns	ns
*iso*14	0.19 ± 0.04 A	0.13 ± 0.01 B	0.19 ± 0.02 A	0.17 ± 0.00 A	*	***	*
*iso*15	0.34 ± 0.07 A	0.24 ± 0.01 B	0.31 ± 0.02 A	0.23 ± 0.01 B	ns	***	ns
*anteiso*15	1.37 ± 0.27 A	0.53 ± 0.02 B	0.65 ± 0.04 B	0.52 ± 0.01 B	***	***	***
*iso*16	0.39 ± 0.08 A	0.27 ± 0.01 B	0.41 ± 0.04 A	0.30 ± 0.01 B	ns	***	ns
*iso*17	0.03 ± 0.06 B	0.02 ± 0.00 B	0.01 ± 0.00 B	0.23 ± 0.03 A	***	***	***
*anteiso*17	0.87 ± 0.11 A	0.26 ± 0.14 D	0.58 ± 0.06 B	0.39 ± 0.02 C	*	***	***
Total BCFAs	3.24 ± 0.50 A	1.50 ± 0.13 C	2.20 ± 0.16 B	1.88 ± 0.02 B	**	***	***
Total OBCFAs	4.97 ± 0.78 A	3.31 ± 0.11 C	4.39 ± 0.19 B	3.71 ± 0.04 C	ns	***	**
Monounsaturated Fatty Acids (MUFAs)							
14:1	0.89 ± 0.36 AB	1.14 ± 0.09 A	0.87 ± 0.10 B	0.96 ± 0.05 AB	ns	*	ns
*trans*, 16:1	0.10 ± 0.09	0.12 ± 0.01	0.08 ± 0.01	0.06 ± 0.00	ns	ns	ns
*cis*, 16:1	0.64 ± 0.50 B	1.55 ± 0.29 A	1.52 ± 0.18 A	1.24 ± 0.15 A	*	**	***
*trans*9, 18:1	0.31 ± 0.07	0.39 ± 0.15	0.37 ± 0.03	0.34 ± 0.03	ns	ns	ns
*trans*11, 18:1	1.97 ± 1.73	2.03 ± 0.33	1.26 ± 0.21	1.32 ± 0.011	ns	ns	ns
*cis*9, 18:1	24.64 ± 3.59 A	17.88 ± 0.57 B	22.22 ± 0.93 A	19.31 ± 0.45 B	ns	***	**
*cis*11, 18:1	0.56 ± 0.16 A	0.55 ± 0.05 A	0.50 ± 0.08 AB	0.40 ± 0.02 B	**	ns	ns
20:1 n9	0.06 ± 0.02	0.05 ± 0.01	0.06 ± 0.01	0.05 ± 0.01	ns	ns	ns
22:1 n9	0.03 ± 0.02	0.04 ± 0.03	0.03 ± 0.01	0.03 ± 0.00	ns	ns	ns
Total MUFAs	29.21 ± 4.34 A	23.75 ± 0.98 B	26.89 ± 0.88 A	23.71 ± 0.46 B	ns	***	ns
Polyunsaturated Fatty Acids (PUFAs)							
t9t12, 18:2 n6	0.01 ± 0.01 A	0.05 ± 0.08 A	0.23 ± 0.02 B	0.21 ± 0.02 B	***	ns	ns
c9t12, 18:2 n6	0.02 ± 0.01 B	0.03 ± 0.01 AB	0.04 ± 0.02 A	0.04 ± 0.02 AB	**	ns	ns
t9c12, 18:2 n6	0.04 ± 0.03	0.04 ± 0.01	0.03 ± 0.01	0.03 ± 0.00	ns	ns	ns
c9c12, 18:2 n6	1.38 ± 0.49 C	2.73 ± 0.32 A	2.25 ± 0.15 B	2.80 ± 0.08 A	**	***	**
18:3 n6	0.02 ± 0.01 AB	0.03 ± 0.02 A	0.02 ± 0.01 B	0.0 ± 0.01 B	*	ns	ns
18:3 n3	0.62 ± 0.17	0.79 ± 0.19	0.73 ± 0.13	0.70 ± 0.03	ns	ns	ns
c9t11–18:2	1.12 ± 0.71 AB	1.31 ± 0.12 A	0.78 ± 0.12 B	0.73 ± 0.04 B	**	ns	ns
20:2	0.02 ± 0.01	0.05 ± 0.03	0.03 ± 0.00	0.03 ± 0.00	ns	ns	ns
20:3 n6	0.07 ± 0.02 B	0.13 ± 0.05 A	0.10 ± 0.01 AB	0.10 ± 0.00 AB	ns	**	**
20:4 n6	0.13 ± 0.02 B	0.16 ± 0.01 B	0.14 ± 0.01 B	0.14 ± 0.00 AB	ns	**	**
20:3 n3	0.01 ± 0.01	0.01 ± 0.01	0.01 ± 0.00	0.01 ± 0.00	ns	ns	ns
20:5 n3	0.06 ± 0.02	0.06 ± 0.02	0.06 ± 0.01	0.05 ± 0.00	ns	ns	ns
22:5 n3	0.09 ± 0.06	0.10 ± 0.02	0.10 ± 0.01	0.08 ± 0.01	ns	ns	ns
Total PUFAs	3.60 ± 0.93 C	5.49 ± 0.29 A	4.51 ± 0.24 B	4.93 ± 0.12 AB	ns	***	**
Total n3 PUFAs	0.79 ± 0.21	0.96 ± 0.24	0.90 ± 0.14	0.84 ± 0.03	ns	ns	ns
Total n6 PUFAs	1.67 ± 0.50 C	3.17 ± 0.22 AB	2.80 ± 0.16 B	3.33 ± 0.11 A	***	***	***
Indexes							
PUFAs/SFAs	0.06 ± 0.02 B	0.08 ± 0.00 A	0.07 ± 0.00 AB	0.07 ± 0.00 AB	ns	**	*
n6/n3	2.20 ± 0.80 C	3.46 ± 0.69 AB	3.16 ± 0.48 B	3.98 ± 0.15 A	**	***	ns
DI ^1^	0.09 ± 0.02	0.08 ± 0.01	0.08 ± 0.01	0.08 ± 00	ns	ns	ns

^1^ DI—desaturase index, calculated as 14:1/(14:0 + 14:1), suggested as the best indicator for Δ9-desaturase activity [35]. The significance obtained in the multifactorial analysis (S—season, F—farm, S × F—interaction) are presented (* *p* < 0.05, ** *p* < 0.01, *** *p* < 0.001). Average values associated with different letters (A, B, C, D) on the same row are statistically different.

**Table 4:** Pairwise correlation matrix between fiber-related parameters (NDF, ADF and F:C ratio) of the diet and the percentages of FAs detected in milk. Only significant (* *p* < 0.05, ** *p* < 0.01, *** *p* < 0.001) correlations are reported.

FA	Diet.NDF		Diet.ADF		F:C Ratio	
	Correlation Coefficient	*sign*	Correlation Coefficient	*sign*	Correlation Coefficient	*sign*
6:0	−0.4632	**	−0.5716	**	−0.4585	**
8:0	−0.699	***	−0.7464	***	−0.6903	***
10:0	−0.8094	***	−0.8296	***	−0.7797	***
12:0	−0.8076	***	−0.8107	***	−0.7515	***
14:0	−0.8101	***	−0.8306	***	−0.7626	***
16:0	−0.4064	*	−0.5001	**		ns
18:0	0.535	**	0.6184	**	0.3569	*
20:0	0.6408	**	0.7656	***	0.579	**
22:0	0.5596	**	0.7062	***	0.573	**
24:0	0.4687	**	0.6019	**	0.521	**
SFA	−0.686	***	−0.746	***	−0.5952	**
13:0	−0.5719	**	−0.521	**	−0.4094	*
15:0	−0.516	**	−0.3702	*	−0.6746	***
17:0	0.794	***	0.8788	***	0.7981	***
*iso*14	0.5696	**	0.6079	**	0.4239	*
*iso*15	0.7487	***	0.8459	***	0.7178	***
*anteiso*15	0.7539	***	0.7586	***	0.927	***
*iso*16	0.7349	***	0.7689	***	0.517	**
*iso*17	−0.478	**	−0.5356	**	−0.4515	*
*anteiso*17	0.8764	***	0.8825	***	0.8641	***
BCFAs	0.8184	***	0.8302	***	0.881	***
OBCFAs	0.7803	***	0.8489	***	0.755	***
14:1	−0.4955	**	−0.5035	**		ns
*cis*, 16:1	−0.6133	**	−0.4999	**	−0.6802	***
*cis*9, 18:1	0.8656	***	0.8404	***	0.7397	***
*cis*11, 18:1		ns		ns	0.376	*
MUFAs	0.761	***	0.7962	***	0.6888	***
t9t12, 18:2 n6		ns		ns	−0.5682	**
c9t12, 18:2 n6		ns		ns	−0.418	*
c9c12, 18:2 n6	−0.7971	***	−0.8484	***	−0.9023	***
20:0	−0.6655	***	−0.6302	**	−0.6721	***
20:3 n6	−0.6809	***	−0.6755	***	−0.7917	***
20:4 n6	−0.462	**	−0.5687	**	−0.4728	**
20:5 n3	0.5192	**	0.528	**	0.3586	*
PUFAs	−0.7067	***	−0.6561	***	−0.749	***
PUFAs/SFAs	−0.4921	**	−0.4132	*	−0.5477	**
n6	−0.7798	***	−0.8173	***	−0.9273	***
n6/n3	−0.7108	***	−0.7938	***	−0.8208	***

## Data Availability

Not applicable.
